# Mortality among British Columbians testing for hepatitis C antibody

**DOI:** 10.1186/1471-2458-13-291

**Published:** 2013-04-02

**Authors:** Amanda Yu, John J Spinelli, Darrel A Cook, Jane A Buxton, Mel Krajden

**Affiliations:** 1 BC Centre for Disease Control, 655 West 12th Avenue, Vancouver, BC, V5Z 4R4, Canada; 2 School of Population & Public Health, University of British Columbia, Vancouver, BC, Canada; 3 BC Cancer Agency, Vancouver, BC, Canada; 4 Department of Pathology & Laboratory Medicine, University of British Columbia, Vancouver, BC, Canada

**Keywords:** Hepatitis C virus, Mortality, Chronic hepatitis, Injection drug use, Data linkage

## Abstract

**Background:**

Hepatitis C virus (HCV) infection is a major preventable and treatable cause of morbidity and mortality. The ability to link population based centralized laboratory HCV testing data with administrative databases provided a unique opportunity to compare mortality between HCV seronegative and seropositive individuals. Through the use of laboratory testing patterns and results, the objective of this study was to differentiate the viral effects of mortality due to HCV infection from risk behaviours/activities that are associated with acquisition of HCV infection.

**Methods:**

Serological testing data from the British Columbia (BC) Centre for Disease Control Public Health Microbiology and Reference Laboratory from 1992–2004 were linked to the BC Vital Statistics Agency death registry. Four groups of HCV testers were defined by their HCV antibody (anti-HCV) testing patterns: single non-reactive (SNR); serial multiple tested non-reactive (MNR); reactive at initial testing (REAC); and seroconverter (SERO) (previously seronegative followed by reactive, a marker for incident infection). Standardized mortality ratios (SMRs) were calculated to compare the relative risk of all cause and disease specific mortality to that of the BC population for each serological group. Time dependent Cox proportional hazard regression was used to compare hazard ratios (HRs) among HCV serological groups.

**Results:**

All anti-HCV testers had higher SMRs than the BC population. Referent to the SNR group, the REAC group had higher risks for liver (HR: 9.62; 95% CI=8.55-10.87) and drug related mortality (HR: 13.70; 95% CI=11.76-16.13). Compared to the REAC group, the SERO group had a lower risk for liver (HR: 0.53; 95% CI=0.24-0.99), but a higher risk for drug related mortality (HR: 1.54; 95% CI=1.12-2.05).

**Conclusions:**

These findings confirm that individuals who test anti-HCV positive have increased mortality related to progressive liver disease, and that a substantial proportion of the mortality is attributable to drug use and risk behaviours/activities associated with HCV acquisition. Mortality reduction in HCV infected individuals will require comprehensive prevention programming to reduce the harms due to behaviours/activities which relate to HCV acquisition, as well as HCV treatment to prevent progression of chronic liver disease.

## Background

Hepatitis C virus (HCV) infection is associated with significant morbidity and mortality. World-wide, approximately 170 million people are infected with HCV, including 243,000 to 300,000 Canadians and 60,000 British Columbians [1-3]. About 25% of infected individuals spontaneously clear infection and 75% become chronically infected [4,5]. Within 20 to 30 years of infection, approximately 10% to 40% of HCV-infected individuals will develop cirrhosis [6], and about 1% to 5% will develop hepatocellular carcinoma [7]. End stage liver disease caused by HCV infection is the primary reason for liver transplantation in Western Europe and North America [8-11]. Most new HCV infections occur in people who inject drugs (IDU) [1], whose risk activities may result in mortality unrelated to HCV infection, and for this population, the risk of death from drug related causes is greater than from liver related causes [12-15]. HCV treatment has been shown to significantly reduce morbidity and mortality [16-18]. Approximately 50% of individuals who are able to tolerate interferon/ribavirin therapies achieve sustained virological response. The addition of protease inhibitors improves curability to about 65% to 75% and the large number of antiviral agents in the therapeutic pipeline will likely result in cure rates of greater than 90% [18].

We examined the HCV-attributable disease burden by estimating all cause and disease specific mortality among individuals who underwent HCV serological testing between 1992 and 2004 in British Columbia (BC), Canada. We also differentiated mortality due to HCV infection from risk activities associated with HCV acquisition.

## Methods

### Study design

The study is a retrospective cohort study that involves secondary data analysis based on linked administrative databases. A cohort of individuals who underwent serological testing for anti-HCV from April 1992 to July 2004 at the BC Centre for Disease Control (BCCDC) Public Health Microbiology and Reference Laboratory were linked to the BC Vital Statistics Agency death registry and the BC Ministry of Health (MoH) Registration & Premium Billing files. The exposure variable, the HCV serological group, was defined based on anti-HCV testing patterns and results. The outcome variables, survival time and underlying cause of death, were obtained from the linked death certificate data.

### Study population

**Figure 1:**
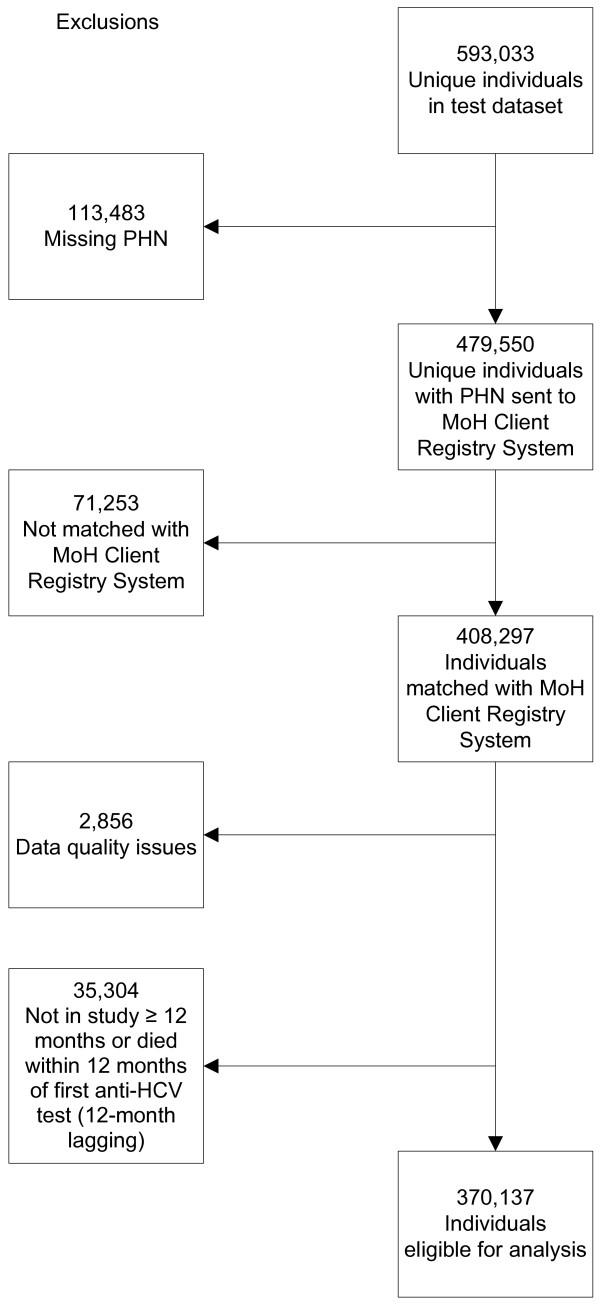
**Study population.** PHN, personal health number; MoH, Ministry of Health.

To be eligible for the study, individuals had to have at least one anti-HCV test between 1992 and 2004 and a valid personal health number (PHN), which is available to anyone who has resided in BC for at least three months and indicates insurance coverage under the BC population Medical Services Plan (MSP). The HCV test dataset contained a total of 593,033 individuals (Figure 1). Unique individuals with a PHN were sent for matching to the BC MoH Client Registry System, the central demographics file for BC residents insured by MSP. Data quality exclusions included situations such as the first available test date was after the date of death or before the date of birth; the date of last registration with MSP was before the test date; the individual was less than one year of age and HCV antibodies may have been maternally acquired; or the individual was more than 100 years of age (n=6), as there were concerns about errors in dates. In order to limit the impact of notification bias from individuals with co-morbid illnesses being tested more often, and persons who died being more likely to have been hospitalized and tested for HCV, the start date for entry to each HCV serological group was delayed for twelve months, i.e., 12-month lagging [19,20]. A total of 370,137 individuals who had at least one anti-HCV test from 1992 to 2004 and remained alive for at least twelve months after their first anti-HCV test were eligible for analysis (Figure 1).

The observation period was the date an individual entered a serological group until: 1) they became part of a different group; 2) they ceased to be registered with MSP; 3) they died; or 4) the study period ended. Individuals who ceased to be registered (usually as a result of leaving the province) and who did not re-register with MSP during the study period were censored at the date of last registration.

### Data sources

The HCV test dataset included all anti-HCV tests performed between April 1992 and July 2004 at the BCCDC Public Health Microbiology and Reference Laboratory, which performs about 95% of anti-HCV testing in BC [21]. HCV testing data were deterministically linked with the MoH administrative databases by Population Data BC, which houses longitudinal person-specific health data on BC residents [22]. The MSP Registration & Premium Billing files were used to determine subject age and sex and date of last registration. All deaths of BC residents registered in the province are captured in the death registry maintained by the BC Vital Statistics Agency. Date of death was used to determine survival time for time to event analysis. Underlying causes of death were coded according to the tenth edition of the International Classification of Diseases (ICD-10). All personally identifiable information was removed from the linked dataset prior to analysis.

### Time dependent HCV serological groups

In order to best represent changes in HCV serological status over time, four time dependent HCV serological groups were defined: 1) single non-reactive (SNR), beginning at the first non-reactive anti-HCV test and ending when a second test was identified; 2) multiple tested non-reactive (MNR), beginning at the second non-reactive test and continuing until a reactive result was identified; 3) anti-HCV reactive at initial testing (REAC) and 4) seroconverter (SERO), beginning when an individual had a reactive result preceded by one or more non-reactive results. No time limit was applied between test dates for defining the MNR and SERO groups, except that the tests had to occur between 1992 and 2004, and had to meet the 12-month lagging period from the first test. The median time between the last negative and first positive tests for the SERO group was 74 weeks (interquartile range; 33, 153). These testing patterns and results were also used as indicators of risk. The SNR group may have had a risk such as history of transfusion prior to screening the blood supply, or they may have been tested for diagnostic work-up or insurance purposes. However, since they had no subsequent tests, they probably had few, if any, ongoing risks. Because of the multiple testing, the MNR group likely had ongoing risks such as IDU or renal dialysis. The REAC group may have been tested because of symptoms of liver disease, or because of risk factors, and likely includes most chronic HCV cases. The SERO group likely had current or ongoing risks, typically IDU. Serological group classification was time dependent and individuals could fall into more than one group, e.g., individuals who had multiple non-reactive anti-HCV results would be in the SNR group at the time of their first test and in the MNR group for their subsequent tests. When a reactive test result occurred, they would become part of the SERO group.

### Statistical methods

Standardized mortality ratios (SMR), observed deaths divided by expected deaths, were calculated to compare all cause and disease specific mortality for each serological group with the BC population; 95% confidence intervals were calculated by Byar’s approximation [23]. Observed deaths were obtained by sex and year by summarizing the number of deaths for each five year age group for each serological group in the study cohort from 1992 to 2004. Expected deaths were obtained by multiplying the calculated person years for each 5-year age stratum in each serological group by the BC population death rate for the respective 5-year age stratum. The same calculation was applied for each disease specific underlying cause of death when calculating a disease specific SMR.

Since assignment of individuals to the HCV serological groups may change over time, the time dependent Cox proportional hazards model [24] was used to estimate the hazard ratios (HR) for mortality between groups, adjusted for age and sex. Survival time was defined as the time of the first group entry until the date of death or last follow-up. Profile likelihood was used to calculate 95% confidence intervals [25]. HRs were calculated for: 1) MNR vs. SNR, to compare the ongoing risk group (MNR) to the lowest risk group (SNR); 2) REAC vs. SNR, to compare the group containing most chronic infections to the lowest risk group; 3) SERO vs. SNR, to compare seroconverters to the lowest risk group; 4) SERO vs. MNR, to compare seropositive to seronegative individuals with ongoing risk; 5) SERO vs. REAC, to compare mortality risk due to activities associated with HCV acquisition vs. the risk resulting from chronic infection. Due to the small number of observed deaths in the SERO group, the REAC and SERO groups were combined to form the HCV positive group for some analyses. Time dependent Cox proportional hazards models were fit to overall mortality and specific causes of death a priori thought to be related to HCV. Models were not applied for causes of death with five or fewer observed deaths in the HCV positive group due to the lack of statistical power. Schoenfeld residuals on functions of time were plotted and examined for each cause of death and for each group comparison [26]. With 12-month lagging, no substantial departures from the proportional hazards were observed.

Interactions between HCV serological groups and age and sex were explored to determine if the age and sex interaction effects were statistically significant (p < 0.01). If the interaction effects were statistically significant, we rejected the hypothesis that the association (HR) did not differ between strata and separate HRs were calculated for each age group or sex, or both. All analyses were performed with SAS Version 9.2 (Statistical Analysis Software, Cary, NC, USA).

### Ethics

The study was conducted in accordance with the Helsinki Declaration of 1975 and was approved by the Clinical Research Ethics Boards at the University of British Columbia and the BC Cancer Agency.

## Results

Baseline demographics for the HCV serological groups are shown in Table 1. Approximately two thirds of the HCV positive group, which includes the REAC and SERO groups, were male. Females were more likely to be tested than males (55% vs. 45%) and females were tested more frequently. The REAC group was older than the SERO group (mean ages 43.4 and 34.1, respectively).

**Table 1:** Baseline demographics for HCV serological groups

**Serological group**^**1**^	**SNR**	**MNR**	**REAC**	**SERO**
n (all)	375,752	90,136	29,689	2,834
n (12 month lagging)^2^	342,325	73,476	27,812	2,408
Person years	1,132,262	216,828	17,780	1,332
Mean age at entry (SD)	41.6 (17.04)	40.6 (15.01)	43.4 (11.48)	34.1 (9.67)
Male (%)	150,178 (44%)	31,457 (43%)	17,780 (64%)	1,332 (55%)
n deaths (%)	12,777 (3.7%)	2,664 (3.6%)	2,705 (9.7%)	119 (4.9%)

*Data sources*: *BCCDC Public Health Microbiology and Reference Laboratory*, *BC Ministry of Health Services*, *and BC Vital Statistics Agency*.

SNR=single non-reactive; MNR=multiple non-reactive; REAC=reactive at first test; SERO=seroconverter.

^1^The serological groups are not mutually exclusive, i.e., over time an individual may fall into more than one group.

^2^The N for 12 month lagging accounts for removal of those subjects who were not followed for a minimum of 12 months or who died within 12 months of their first anti-HCV test. Those subjects excluded by 12 month lagging did not form part of the standardized mortality and hazard ratio analyses.

The SMRs for all cause and disease specific mortality are shown in Tables 2 and 3. All serological groups had significantly higher all cause mortality than the BC population, with the highest SMR in the SERO group, followed by the REAC, MNR and SNR groups. The SMR for liver related mortality was highest in the REAC group, followed by the SERO group, whereas the reverse was true for drug related mortality. In addition, the SMR for mortality due to mental and behavioural disorders was highest in the SERO group (Table 3).

**Table 2:** **Standardized mortality ratios for HCV serological groups vs**. **BC population**

		**SNR (n=342,325)**	**MNR (n=73,476)**	**REAC (n=27,812)**	**SERO (n=2,408)**	**All HCV +ve (n=30,220)**
ICD-10	Cause of death	SMR (95% CI)	SMR (95% CI)	SMR (95% CI)	SMR (95% CI)	SMR (95% CI)
A00-R99; V01-Y98	All	1.44 (1.41 - 1.46)	2.59 (2.49 - 2.69)	4.68 (4.50 - 4.86)	10.13 (8.39 - 12.12)	4.78 (4.61 - 4.96)
B15-B19; B942; C22; K70-K76	Liver related	3.00 (2.77 - 3.24)	8.44 (7.47 - 9.50)	23.93 (22.03 - 25.95)	14.77 (6.36 - 29.09)	23.73 (21.86 - 25.72)
B15-B19; B942	Viral hepatitis	1.04 (0.70 - 1.49)	2.12 (1.06 - 3.79)	65.32 (58.51 - 72.70)	40.31 (13.06 - 93.93)	64.73 (58.03 - 71.99)
C22	Liver cancer	2.64 (2.26 - 3.07)	4.79 (3.46 - 6.48)	17.31 (14.10 - 21.04)	9.02 (0.23 - 50.23)	17.16 (13.99 - 20.83)
K70-K76	Liver disease	3.68 (3.34 - 4.05)	11.96 (10.43 - 13.65)	11.09 (9.38 - 13.01)	6.52 (0.79 - 23.53)	10.99 (9.31 - 12.88)
K70	Alcoholic	3.64 (3.15 - 4.20)	8.25 (6.50 - 10.33)	10.58 (8.39 - 13.17)	5.45 (0.14 - 30.37)	10.46 (8.30 - 13.00)
K71-K76	Non-alcoholic	3.71 (3.25 - 4.21)	15.65 (13.20 - 18.43)	11.69 (9.11 - 14.77)	8.05 (0.20 - 44.85)	11.62 (9.07 - 14.66)
F11-F16; F19; X40-X44; X60-X64; X85; Y10-Y14	Drug related	1.77 (1.56 - 2.00)	4.54 (3.80 - 5.37)	19.94 (18.31 - 21.69)	36.66 (27.12 - 48.46)	20.72 (19.09 - 22.45)
B20-B24	HIV	1.89 (1.48 - 2.37)	4.08 (2.81 - 5.73)	26.70 (23.49 - 30.22)	35.61 (18.94 - 60.89)	27.03 (23.86 - 30.50)
E10-E14	Diabetes	1.86 (1.69 - 2.03)	5.66 (4.84 - 6.57)	2.21 (1.52 - 3.10)	4.49 (0.11 - 24.99)	2.24 (1.55 - 3.13)
N17-N19; I12-I13; N00-N08	Renal failure	2.41 (2.16 - 2.69)	12.96 (11.12 - 15.03)	4.58 (3.07 - 6.59)	14.85 (0.38 - 82.72)	4.69 (3.16 - 6.70)
C00-C97	Malignant neoplasm	1.34 (1.30 - 1.39)	1.71 (1.57 - 1.86)	2.35 (2.13 - 2.59)	2.03 (0.74 - 4.42)	2.34 (2.12 - 2.58)
C22.0; C22.2-C22.9	Hepatocellular carcinoma	5.34 (4.47 - 6.34)	9.58 (6.67 - 13.32)	36.68 (29.85 - 44.62)	17.04 (0.43 - 94.89)	36.27 (29.54 - 44.07)

*Data sources*: *BCCDC Public Health Microbiology and Reference Laboratory*, *BC Ministry of Health Services*, *and BC Vital Statistics Agency*.

SNR=single non-reactive; MNR=multiple non-reactive; REAC=reactive at first test; SERO=seroconverter; SMR=standardized mortality ratio.

**Table 3:** **Disease specific standardized mortality ratios for HCV serological groups vs**. **BC population**

		**SNR (n=342,325)**	**MNR (n=73,476)**	**REAC (n=27,812)**	**SERO (n=2,408)**	**All HCV +ve (n=30,220)**
ICD-10	Cause of death	SMR (95% CI)	SMR (95% CI)	SMR (95% CI)	SMR (95% CI)	SMR (95% CI)
A00-B99	Infection	1.57 (1.38 - 1.78)	4.36 (3.55 - 5.30)	31.51 (29.08 - 34.09)	32.09 (19.32 - 50.05)	31.53 (29.14 - 34.07)
C00-D48	Neoplasms	1.35 (1.31 - 1.40)	1.72 (1.59 - 1.87)	2.35 (2.14 - 2.59)	1.98 (0.73 - 4.31)	2.35 (2.13 - 2.58)
D50-D89	Blood/immune	1.82 (1.33 - 2.43)	5.18 (2.83 - 8.71)	7.91 (4.09 - 13.84)	36.40 (0.92 - 202.75)	8.41 (4.48 - 14.39)
E00-E90	Endocrine	1.82 (1.67 - 1.97)	5.29 (4.58 - 6.07)	2.07 (1.48 - 2.82)	3.21 (0.08 - 17.87)	2.09 (1.50 - 2.83)
F00-F99	Mental and behavioural	1.16 (1.02 - 1.31)	2.61 (1.99 - 3.37)	6.79 (5.43 - 8.37)	16.01 (4.35 - 40.98)	6.96 (5.60 - 8.55)
G00-G99	Nervous system	1.05 (0.95 - 1.17)	1.49 (1.11 - 1.95)	1.78 (1.21 - 2.53)	6.96 (0.84 - 25.11)	1.87 (1.29 - 2.62)
I00-I99	Circulatory system	1.42 (1.38 - 1.46)	2.22 (2.06 - 2.40)	2.21 (1.98 - 2.45)	6.04 (3.01 - 10.81)	2.25 (2.03 - 2.50)
J00-J99	Respiratory system	1.29 (1.22 - 1.37)	1.70 (1.44 - 1.99)	2.96 (2.46 - 3.53)	5.36 (0.65 - 19.36)	2.98 (2.48 - 3.55)
K00-K93	Digestive system	2.36 (2.20 - 2.53)	7.40 (6.60 - 8.27)	7.97 (6.91 - 9.14)	10.26 (3.33 - 23.92)	8.01 (6.96 - 9.17)
N00-N99	Genitourinary system	1.95 (1.74 - 2.17)	10.47 (8.96 - 12.16)	4.54 (3.14 - 6.34)	12.21 (0.31 - 68.02)	4.62 (3.22 - 6.42)
R00-R99	Other	0.92 (0.68 - 1.22)	3.48 (2.25 - 5.15)	7.43 (5.15 - 10.39)	21.58 (5.87 - 55.24)	7.99 (5.65 - 10.96)
V01-Y98	External	1.44 (1.34 - 1.54)	2.64 (2.34 - 2.98)	8.58 (7.96 - 9.22)	15.25 (11.72 - 19.51)	8.88 (8.28 - 9.52)

*Data sources*: *BCCDC Public Health Microbiology and Reference Laboratory*, *BC Ministry of Health Services*, *and BC Vital Statistics Agency*.

SNR=single non-reactive; MNR=multiple non-reactive; REAC=reactive at first test; SERO=seroconverter; SMR=standardized mortality ratio.

Hazard ratios comparing the mortality risk between the HCV serological groups are shown in Table 4 (and Additional file 1: Tables S1–S2). With respect to all cause mortality, the MNR, REAC and SERO groups had significantly higher mortality risk compared to the SNR group, particularly for younger individuals. The risk of liver related mortality was about ten times higher (HR=9.62; 95% CI=8.55-10.87) for the REAC group vs. the SNR group and about five times higher (HR=5.10; 95% CI=2.30-9.62) for the SERO vs. SNR group. The SERO group had about one half the liver related mortality risk of the REAC group (HR=0.53; 95% CI=0.24-0.99). Drug related mortality risk was significantly higher for both HCV positive groups vs. the SNR group (SERO: HR=20.83; 95% CI=15.15-28.57; REAC: HR=13.70; 95% CI=11.76-16.13). Drug related mortality risk was also significantly higher for the SERO vs. REAC groups (HR=1.54; 95% CI=1.12-2.05). The risk of death from human immunodeficiency virus (HIV) infection was significantly higher in both the REAC and SERO groups vs. SNR, particularly for females. However, the risk of death from HIV infection was not significantly different for the SERO vs. REAC comparison (females: HR=0.64; 95% CI=0.16-1.75; males: HR=0.90; 95% CI=0.44-1.63).

**Table 4:** Hazard ratios between HCV serological groups

		**MNR vs. SNR**	**REAC vs. SNR**	**SERO vs. SNR**	**SERO vs. MNR**	**SERO vs. REAC**
ICD-10	Cause of Death	HR (95% CI)	HR (95% CI)	HR (95% CI)	HR (95% CI)	HR (95% CI)
A00-R99; V01-Y98	All					
	Age <40	2.07 (1.82-2.36)	7.25 (6.54-8.06)	7.52 (5.85-9.52)	3.62 (2.79-4.65)	1.03 (0.80-1.31)
	Age ≥40	1.42 (1.36-1.49)	2.39 (2.27-2.51)	3.36 (2.49-4.41)	2.36 (1.75-3.11)	1.41 (1.04-1.85)
B15-B19; B942; C22; K70-K76	Liver related	2.72 (2.34-3.14)	9.62 (8.55-10.87)	5.10 (2.30-9.62)	1.88 (0.85-3.55)	0.53 (0.24-0.99)
F11-F16; F19; X40-X44; X60-X64; X85; Y10-Y14	Drug related	2.62 (2.11-3.25)	13.70 (11.76-16.13)	20.83 (15.15-28.57)	8.00 (5.71-11.11)	1.54 (1.12-2.05)
B20-B24	HIV related					
	Female	3.03 (0.90-9.52)	83.33 (40.00-200.00)	52.63 (11.49-200.00)	17.54 (3.62-71.43)	0.64 (0.16-1.75)
	Male	1.77 (1.11-2.74)	12.05 (9.09-16.13)	10.87 (5.24-20.41)	6.17 (2.84-12.35)	0.90 (0.44-1.63)

*Data sources*: *BCCDC Public Health Microbiology and Reference Laboratory*, *BC Ministry of Health Services*, *and BC Vital Statistics Agency*.

MNR=multiple non-reactive; SNR=single non-reactive; REAC=reactive at first test; SERO=seroconverter; HR=hazard ratio.

## Discussion

This study examined all cause and disease specific mortality among individuals tested for HCV antibody in BC from 1992 to 2004. A unique feature of the study was the use of a population based central laboratory database to identify HCV-tested individuals (both positive and negative results) and to correlate the risk of death with serological results and testing patterns. To our knowledge this is the first study to use time dependent HCV serological groups to help stratify infection status and mortality risks. While the risk of death among all individuals who underwent HCV antibody testing was significantly higher than the BC population, the serological groupings helped to differentiate mortality which was likely related to risk activities associated with HCV acquisition from mortality associated with HCV related chronic liver disease.

Our findings confirm that mortality among seroconverters was more likely to be drug related. This is consistent with other published studies which demonstrated no increase in liver related mortality among seroconverters during the first decade after infection [27,28], whereas accidental deaths, including drug overdose, contributed significantly [27]. This is expected because acute HCV infection rarely results in fulminant liver failure [4], and decades are required to develop cirrhosis [6]. In contrast, individuals whose first anti-HCV test was reactive were more likely to have chronic HCV infection and had higher risk of liver related mortality, partly as a result of being older and having survived their substance use. Drug use was also an important mortality contributor in the REAC group, and in addition, both the REAC and SERO groups had increased HIV related mortality.

While it could be argued that the designation of SNR, MNR, REAC and SERO groups is arbitrary, the grouping assumptions are supported by the data. The SERO group was the best characterized group because the date of a previous negative anti-HCV test was known. The REAC group was likely heterogeneous and included persons who may have been infected in the past from contaminated blood products, past or present IDU or other risk factors [29]. The lack of previous serology prevents their identification as a seroconverter. Persons in the REAC group had the highest liver related death rate. However, it is recognized that about 25% of HCV-infected individuals will spontaneously clear infection and remain antibody reactive [4] and such individuals are not known to be at increased risk of HCV related liver disease [30]. In contrast, seronegative individuals who tested only once likely had some baseline risks which led to serological testing. Therefore, the SNR group represents a relatively conservative comparator group given that their mortality risks were only slightly higher than the general BC population. Multiple negative testers likely had ongoing risks. For example, they were more likely to die of renal failure, diabetes and both alcoholic and non-alcoholic liver disease.

Our results are consistent with other population based studies, summarized in Table 5, from Australia [12], Sweden [13] and Scotland [15], which reported SMRs 17- to 46-fold higher for liver related and 19- to 37-fold higher for drug related mortality among HCV-infected persons vs. the general population. As expected, the mean age of the SERO group was significantly lower than the REAC group, which likely reflects a larger proportion of younger IDUs in the SERO group. Of note, 83% of individuals in BC who seroconverted within the previous twelve months and who could be contacted reported a history of IDU [31].

**Table 5:** Comparison of standardized mortality ratios with other studies

**Author**	**Country**		**All cause SMR (95% CI)**	**Liver related SMR (95% CI)**	**Drug related SMR (95% CI)**
Amin [12] (2006)	Australia	HCV mono-infected	3.1 (3.0-3.2)	16.8 (15.4-18.3)	19.3 (18.1-20.5)
		HCV/HBV co-infected	5.6 (4.8-6.6)	32.9 (23.1-46.7)	24.7 (18.2-33.5)
Duberg [13] (2008)	Sweden	HCV mono-infected	5.8 (5.6-6.0)	35.5 (32.9-38.3)	20.7 (18.9-22.7)
		HCV/HBV co-infected	8.5 (7.3-9.8)	46.2 (31.5-62.3)	27.6 (19.6-39.6)
McDonald [15] (2008)	Scotland	HCV mono-infected	4.9 (4.6-5.1)	20.0 (17.9-22.2)	23.5 (21.3-25.7)
		HCV/HIV co-infected	32.9 (29.2-37.0)	34.8 (23.3-50.0)	36.6 (25.2-51.4)
Yu (2012)	Canada	All HCV-infected	4.8 (4.6-5.0)	23.7 (21.9-25.7)	20.7 (19.1-22.5)
		REAC	4.7 (4.5-4.9)	23.9 (22.0-26.0)	19.9 (18.3-21.7)
		SERO	10.1 (8.4-12.1)	14.8 (6.4-29.1)	36.7 (27.1-48.5)

SMR=standardized mortality ratio; HCV=hepatitis C virus; HBV=hepatitis B virus; HIV=human immunodeficiency virus; REAC=reactive at first test; SERO=seroconverter.

A recent study in the United States by El-Kamary et al. [32] utilized data from the National Health and Nutrition Examination Survey (NHANES) to examine mortality among HCV-uninfected, HCV antibody positive/HCV RNA negative, and HCV chronically infected individuals. This study used mortality rate ratios that showed all cause mortality among chronically infected individuals was more than twice that of HCV negative individuals and that 57.8% of the mortality was attributable to HCV. While our study demonstrated an increased drug related mortality in association with HCV infection, El-Kamary et al. [32] did not find a significant association between HCV status and non-liver related deaths. This may reflect differences in the underlying risk factors in the populations studied, i.e., the NHANES-based study reflects a random population sample which was then tested for HCV, whereas our study involved individuals who underwent diagnostic HCV testing.

Co-infections with HIV and hepatitis B virus (HBV) are common in HCV-infected individuals, and co-infected individuals have increased mortality relative to HCV mono-infected individuals [12-15]. Although we were unable to differentiate HCV mono-infected individuals from those co-infected, the HCV positive group (REAC and SERO groups combined) would be expected to include HBV and HIV co-infected individuals. HIV and HCV prevalence rates among BC IDUs have been reported as 17% [33] and 83% [34] respectively, and among HIV positive individuals in BC, the HCV co-infection rate has been estimated to be between 27% and 53% [35]. Unfortunately, the HIV status was unknown unless the individual died of HIV related causes. The SMRs for all cause, liver and drug related mortality for our HCV positive group were, however, similar to other HIV co-infected populations [15].

The time dependent Cox proportional hazard modelling revealed an 83-fold higher HIV related mortality for women in the REAC group relative to SNR women, whereas REAC men displayed a 12-fold higher mortality relative to SNR men. This may reflect the lack of access to care by highly marginalized women who are then more likely to die of HIV related causes.

This study has important public health implications. In the short term, younger drug users, here represented by the seroconverters, would likely benefit more from harm reduction, including needle distribution and methadone maintenance programs to reduce the risk of drug related mortality, than from antiviral treatment when their risk of liver related mortality is low [36]. In contrast, individuals with chronic HCV infection would benefit from harm reduction programs and antiviral treatment to reduce the risk of drug related mortality and progressive liver disease. These findings are consistent with a recent HCV costing analysis in BC where substantial HCV related direct healthcare costs could be ascribed to mental health, addictions and associated risk activities which lead to HCV acquisition, as well as the consequences of progressive liver disease [21]. Of note, early treatment of IDUs has been shown to be cost-effective [37]. Since most onward HCV transmission involves IDU, virological cure could potentially reduce incident HCV infections.

The study has several limitations. Approximately 20% of individuals in the laboratory dataset had no PHN and could not be deterministically linked, which could lead to exclusion of vulnerable individuals, e.g., with no fixed address, who have been shown to have higher HCV prevalence and morbidity/mortality risks compared to the general population [1]. As with all administrative data linkages, the ability to control for confounders such as HCV transmission risks, co-morbidities or social determinants of health, which may impact access to testing, is limited. Misclassification of seroconverters within the REAC group would occur if a recently infected individual had no prior HCV test, but the SERO vs. REAC comparison was considered a reasonable surrogate for comparing incident vs. chronic HCV infection. All of these limitations could lead to underestimation of SMR and HR in our study. Seriously ill individuals or recipients of unscreened blood products in the past are more likely to be tested, and our attempt to compensate for potential notification bias by applying a twelve month lagging period to study enrolment may have been insufficient. We did not analyze HCV RNA results which were available for only a limited number of individuals. Therefore, individuals who had cleared their infection would have been classified as incident or chronic infections based on their anti-HCV test results. Since individuals who spontaneously clear their HCV infection are not known to be at risk of progressive liver disease [30], our SMR and HR estimates for mortality due to chronic HCV infection, especially for liver related mortality, are likely conservative. Different anti-HCV screening tests were used during the study period (second generation from 1992 to 1997 and third generation from 1997 to 2004). However, both assays have good sensitivity and specificity [38] so this is unlikely to significantly affect diagnostic accuracy. The death register data are considered reliable as registration is a mandatory requirement, subject to federal/provincial standards and coded according to ICD-10 classification. However, since only 7.1% of all deaths in BC (2009 data) had an autopsy [39], the listed underlying cause of death may not be completely reliable. Deaths attributable to viral hepatitis primarily result from chronic liver disease and liver failure, and viral hepatitis may not consistently be listed as the underlying cause of death, leading to underestimation of deaths due to chronic viral hepatitis. In addition, deaths which occurred outside of BC were not captured in the analysis.

The strengths of this study include the large sample size, cohort design, and longitudinal population based serological data. The large sample size allowed us to more precisely measure the effect of mortality attributable to HCV infection than smaller studies. The longitudinal serological data with HCV seropositive and seronegative results enabled the identification of incident HCV cases and multiple non-reactive testers. This information helped to differentiate HCV related mortality due to progressive liver disease from the risk behaviours/activities that are associated with HCV acquisition.

## Conclusions

We found excess mortality due to progressive liver disease and risk activities related to HCV acquisition among individuals who test anti-HCV positive. From a policy perspective, reduction of HCV related mortality requires comprehensive prevention and harm reduction programming and improved treatment uptake to reduce the consequences of chronic HCV infection, which include progressive liver disease and other extra-hepatic illnesses.

## Abbreviations

HCV: hepatitis C virus; BC: British Columbia; SNR: Single non-reactive; MNR: Multiple tested non-reactive; REAC: Anti-HCV reactive at initial testing; SERO: Seroconverter; SMR: Standardized mortality ratio; HR: Hazard ratio; IDU: People who inject drugs; BCCDC: British Columbia Centre for Disease Control; MoH: Ministry of Health; PHN: Personal Health Number; MSP: Medical Services Plan; ICD-10: International Classification of Diseases, 10^th^ Revision; HIV: Human immunodeficiency virus; NHANES: National Health and Nutrition Examination Survey; HBV: Hepatitis B virus.

## Competing interests

The authors declare that they have no competing interests.

## Authors’ contributions

AY, JJS and MK conceived and designed the study. AY acquired the data, performed the statistical analyses and interpreted the data. AY and DAC drafted the manuscript. JJS, JAB and MK supervised the study. MK obtained grant funding. All authors provided critical revision of the manuscript for important intellectual content and gave final approval of the version to be published.

## Pre-publication history

The pre-publication history for this paper can be accessed here:

http://www.biomedcentral.com/1471-2458/13/291/prepub

## Supplementary Material

Additional file 1: Table S1 Hazard ratios for a priori mortality endpoints. **Table S2.** Hazard ratios for disease specific mortality endpoints. Click here for file
