# Supplementary material for: Mortality among British Columbians testing for hepatitis C antibody
Source: BMC Public Health. 2013 Apr 2;13:291. doi: 10.1186/1471-2458-13-291 (PMC3626540; doi:10.1186/1471-2458-13-291)
Supplement: Additional file 1: Table S1 — Hazard ratios for a priori mortality endpoints. Table S2. Hazard ratios for disease specific mortality endpoints. [file 1471-2458-13-291-S1.doc]

# Supplementary Material

Supplementary Table 1. Hazard ratios for *a priori* mortality endpoints

|  |  | SNR | MNR | HCV +ve | MNR vs. SNR | | HCV +ve vs. SNR | | HCV +ve vs. MNR | |
| --- | --- | --- | --- | --- | --- | --- | --- | --- | --- | --- |
| ICD-10 | Cause of Death | N deaths | N deaths | N deaths | HR | 95% CI | HR | 95% CI | HR | 95% CI |
| A00-R99; V01-Y98 | All | 12,777 | 2,664 | 2,824 |  |  |  |  |  |  |
|  | Age < 40 | 628 | 279 | 531 | 2.07 | (1.82-2.35) | 7.30 | (6.58-8.06) | 3.52 | (3.10-4.00) |
|  | Age ≥ 40 | 12,149 | 2,385 | 2,293 | 1.42 | (1.35-1.48) | 2.40 | (2.28-2.52) | 1.69 | (1.59-1.80) |
| B15-B19; B942; C22; K70-K76 | Liver related | 632 | 273 | 594 | 2.73 | (2.35-3.16) | 9.62 | (8.47-10.75) | 3.51 | (3.02-4.08) |
| B15-B19; B942 | Viral hepatitis | 30 | 11 | 340 | 2.09 | (1.00-4.07) | 111.11 | (76.92-166.67) | 52.63 | (30.30-100.00) |
| C22 | Liver cancer | 170 | 42 | 102 | 1.81 | (1.26-2.55) | 7.46 | (5.75-9.71) | 4.13 | (2.87-6.02) |
| K70-K76 | Liver disease | 432 | 220 | 152 |  |  |  |  |  |  |
|  | Age < 40 | 8 | 10 | 11 | 5.88 | (2.58-13.89) | 13.16 | (6.49-29.41) | 2.25 | (1.16-4.57) |
|  | Age ≥ 40 | 424 | 210 | 141 | 3.16 | (2.65-3.77) | 3.03 | (2.46-3.72) | 0.96 | (0.76-1.20) |
| K70 | Alcoholic | 194 | 76 | 81 | 2.26 | (1.70-2.98) | 3.08 | (2.33-4.03) | 1.36 | (0.98-1.88) |
| K71-K76 | Non-alcoholic | 238 | 144 | 71 |  |  |  |  |  |  |
|  | Age < 40 | <5 | <5 | 7 | 4.57 | (1.08-19.23) | 18.52 | (6.41-66.67) | 4.08 | (1.41-14.71) |
|  | Age ≥ 40 | 234 | 140 | 64 | 4.12 | (3.29-5.15) | 3.13 | (2.30-4.17) | 0.76 | (0.55-1.03) |
| F11-F16; F19; X40-X44; X60-X64; X85; Y10-Y14 | Drug related | 258 | 134 | 596 | 2.60 | (2.10-3.22) | 14.08 | (12.05-16.39) | 5.41 | (4.48-6.58) |
| B20-B24 | HIV related | 73 | 33 | 263 |  |  |  |  |  |  |
|  | Female | 7 | 5 | 63 | 3.04 | (0.90-9.52) | 83.33 | (40.00-200.00) | 27.03 | (11.90-76.92) |
|  | Male | 66 | 28 | 200 | 1.77 | (1.12-2.75) | 12.05 | (9.09-16.13) | 6.80 | (4.63-10.31) |
| E10-E14 | Diabetes | 474 | 170 | 34 | 2.78 | (2.30-3.33) | 1.09 | (0.75-1.53) | 0.39 | (0.27-0.56) |
| N17-N19; I12-I13; N00-N08 | Renal Failure | 326 | 176 | 30 | 5.00 | (4.12-6.06) | 1.68 | (1.12-2.41) | 0.34 | (0.22-0.49) |
| C00-C97 | Malignant neoplasm | 3,416 | 559 | 417 |  |  |  |  |  |  |
|  | Age < 40 | 117 | 47 | 12 | 1.96 | (1.42-2.67) | 1.55 | (1.01-2.29) | 0.79 | (0.49-1.23) |
|  | Age ≥ 40 | 3,299 | 512 | 405 | 1.15 | (1.05-1.27) | 1.63 | (1.46-1.82) | 1.42 | (1.24-1.62) |
| C22.0; C22.2-C22.9 | Hepatocellular carcinoma | 131 | 35 | 101 | 1.90 | (1.27-2.76) | 9.43 | (7.09-12.35) | 4.95 | (3.36-7.46) |

*Data sources: BCCDC Public Health Microbiology and Reference Laboratory, BC Ministry of Health Services, and BC Vital Statistics Agency.*

SNR=single non-reactive; MNR=multiple non-reactive; HR=hazard ratio

**Supplementary Table 2. Hazard ratios for disease specific mortality endpoints**

|  |  | SNR | MNR | HCV +ve | MNR vs. SNR | | HCV +ve vs. SNR | | HCV +ve vs. MNR | |
| --- | --- | --- | --- | --- | --- | --- | --- | --- | --- | --- |
| ICD-10 | Cause of Death | N deaths | N deaths | N deaths | HR | 95% CI | HR | 95% CI | HR | 95% CI |
| A00-B99 | Infection | 239 | 101 | 640 | 2.32 | (1.82-2.93) | 22.73 | (19.23-26.32) | 9.71 | (7.87-12.05) |
| C00-D48 | Neoplasms | 3,527 | 575 | 427 |  |  |  |  |  |  |
|  | Age < 40 | 120 | 48 | 12 | 1.90 | (1.38-2.58) | 1.47 | (0.96-2.16) | 0.77 | (0.48-1.20) |
|  | Age ≥ 40 | 3,407 | 527 | 415 | 1.15 | (1.05-1.27) | 1.63 | (1.46-1.81) | 1.42 | (1.24-1.62) |
| D50-D89 | Blood/immune | 46 | 14 | 13 | 1.99 | (1.02-3.65) | 3.51 | (1.75-6.54) | 1.76 | (0.80-3.85) |
| E00-E90 | Endocrine | 587 | 201 | 41 | 2.61 | (2.20-3.09) | 1.06 | (0.75-1.44) | 0.40 | (0.28-0.56) |
| F00-F99 | Mental and behavioural | 250 | 59 | 91 |  |  |  |  |  |  |
|  | Age < 40 | <5 | 8 | 18 | 4.61 | (1.76-12.35) | 26.32 | (12.66-62.50) | 5.65 | (2.84-12.50) |
|  | Age ≥ 40 | 246 | 51 | 73 | 1.39 | (1.01-1.89) | 2.66 | (1.93-3.61) | 1.91 | (1.29-2.84) |
| G00-G99 | Nervous system | 350 | 53 | 33 | 0.93 | (0.68-1.25) | 1.37 | (0.93-1.95) | 1.46 | (0.93-2.26) |
| I00-I99 | Circulatory system | 4,343 | 681 | 362 |  |  |  |  |  |  |
|  | Age < 40 | 33 | 24 | 26 | 3.28 | (2.09-5.13) | 5.13 | (3.29-7.94) | 1.56 | (0.98-2.49) |
|  | Age ≥ 40 | 4,310 | 657 | 336 | 1.18 | (1.08-1.28) | 1.39 | (1.23-1.55) | 1.18 | (1.03-1.34) |
| J00-J99 | Respiratory system | 1,198 | 151 | 124 |  |  |  |  |  |  |
|  | Age < 40 | 12 | <5 | 9 | 1.78 | (0.63-4.44) | 7.63 | (3.64-16-13) | 4.27 | (1.74-12.05) |
|  | Age ≥ 40 | 1,186 | 147 | 115 | 0.91 | (0.76-1.08) | 1.63 | (1.32-1.99) | 1.79 | (1.39-2.30) |
| K00-K93 | Digestive system | 811 | 311 | 210 |  |  |  |  |  |  |
|  | Age < 40 | 22 | 14 | 14 | 3.47 | (1.94-6.13) | 6.94 | (4.13-11.76) | 2.00 | (1.16-3.52) |
|  | Age ≥ 40 | 789 | 297 | 196 | 2.53 | (2.19-2.92) | 2.80 | (2.36-3.31) | 1.11 | (0.91-1.34) |
| N00-N99 | Genitourinary system | 319 | 171 | 35 | 4.81 | (3.94-5.81) | 2.04 | (1.41-2.87) | 0.43 | (0.29-0.61) |
| R00-R99 | Other | 49 | 25 | 38 | 3.02 | (1.80-4.95) | 7.14 | (4.50-11.24) | 2.36 | (1.41-4.03) |
| V01-Y98 | External | 854 | 271 | 793 |  |  |  |  |  |  |
|  | Age < 40 | 309 | 119 | 299 | 1.86 | (1.52-2.26) | 7.94 | (6.85-9.17) | 4.27 | (3.52-5.21) |
|  | Age ≥ 40 | 545 | 152 | 494 | 1.60 | (1.32-1.94) | 5.15 | (4.48-5.95) | 3.23 | (2.65-3.95) |

*Data sources: BCCDC Public Health Microbiology and Reference Laboratory, BC Ministry of Health Services, and BC Vital Statistics Agency.*

SNR=single non-reactive; MNR=multiple non-reactive; HR=hazard ratio
